# The trajectory of maternal perinatal depressive symptoms predicts executive function in early childhood

**DOI:** 10.1017/S0033291723002118

**Published:** 2023-12

**Authors:** Josephine Power, Stuart Watson, Wai Chen, Andrew Lewis, Marinus van IJzendoorn, Megan Galbally

**Affiliations:** 1 School of Clinical Sciences, Department of Psychiatry, Monash University, Clayton, VIC, Australia; 2 Health Futures Institute, Murdoch University, Murdoch, WA, Australia; 3 Fiona Stanley Hospital, Murdoch, Perth, Australia; 4 Graduate School of Education, University of Western Australia, Perth, Australia; 5 Curtin Medical School, Curtin University, Perth, Australia; 6 Curtin enAble Institute, Curtin University, Perth, Australia; 7 Centre of Healthy Aging, Murdoch University, Perth, Australia; 8 Faculty of Medical Science, Naresuan University, Phitsanulok, Thailand; 9 University of Western Australia Medical School, Perth, WA, Australia; 10 Institute for Health and Wellbeing, Federation University Australia, Mount Helen, VIC, Australia; 11 Erasmus Universiteit Rotterdam, Rotterdam, Netherlands

**Keywords:** child development, executive function, neurodevelopment, perinatal depression

## Abstract

**Background:**

Perinatal maternal depression may affect fetal neurodevelopment directly or indirectly via exposures such as smoking, alcohol, or antidepressant use. The relative contribution of these risk factors on child executive function (EF) has not been explored systematically.

**Methods:**

A prospective pregnancy cohort of 197 women and their children was studied to determine whether maternal depression diagnosis and the trajectory of maternal depressive symptoms (MDSs) from early pregnancy to 12 months postpartum predicts child EF at age 4 (measured using the preschool age psychiatric assessment, NEPSY-II, and Shape School task) using latent growth curve modeling. Indirect effects of smoking, alcohol, and antidepressant use were also formally tested.

**Results:**

Increasing maternal perinatal depressive symptoms over time predicted more inattentive symptoms, poorer switching, and motor inhibition, but not cognitive inhibition. When adjusted for multiple comparison, and after accounting for maternal cognition and education, the association with child inattentive symptoms remained significant. However, diagnosed depression did not predict child EF outcomes. Prenatal exposure to smoking, alcohol, and antidepressants also did not mediate pathways from depressive symptoms to EF outcomes. Our findings were limited by sample size and statistical power to detect outcome effects of smaller effect size.

**Conclusions:**

This study suggests that increasing MDSs over the perinatal period is associated with poorer EF outcomes in children at age 4 – independent of prenatal smoking, drinking, or antidepressant use. Depressive chronicity, severity, and postpartum influences may play crucial roles in determining childhood outcomes of EF.

Maternal perinatal depression affects approximately 12% (Woody, Ferrari, Siskind, Whiteford, & Harris, 2017) of women from conception to 12 months postpartum (NICE, 2021), and is associated with adverse developmental outcomes in children beyond this first year of life (Goodman, 2019; Meaney, 2018; Stein et al., 2014). Evidence indicates an increase in poorer physical, social, psychological, and cognitive outcomes from early childhood, but the underlying mechanisms are unclear (Aktar et al., 2019; Stein et al., 2014). Recent studies propose executive function (EF) as a link between early exposure to maternal depression and at-risk developmental trajectories (Munakata & Michaelson, 2021). Given the role of healthy EF development in later cognitive and self-control functioning, understanding the impact of maternal depression is critical.

EFs are a set of higher cognitive abilities that direct purposeful behavior and co-ordinate mental processes (Lezak, 1982). EF emerges during infancy and involves separable but interconnected capacities for working memory, response inhibition, and shifting of attention (Friedman & Robbins, 2022; Miyake et al., 2000). Development of EF is closely tied to maturation of the prefrontal cortex and is vulnerable to disruption from various prenatal and postnatal exposures. In large observational studies, disruption in EF development is linked to poorer academic performance (Best & Miller, 2010), future physical and mental health, and future socioeconomic status in offspring (Moffit et al., 2011). Meta-analysis of the association between EF and primary school academic performance has revealed a substantial association of *r* = 0.365 (Cortés Pascual, Moyano Muñoz, & Quílez Robres, 2019). The mechanisms underlying these associations are complex, with many potential candidate pathways.

The prenatal and postnatal environments carry distinct developmental risks. Compared with postnatal depression, prenatal depression is less well explored, but might transmit risk through genetic or epigenetic inheritance, inflammatory changes, or hormonal stress responses (Lewis, 2014). Risky behaviors that are known teratogens (DiFranza, Aligne, & Weitzman, 2004; Polanska, Jurewicz, & Hanke, 2015; Streissguth, Barr, & Sampson, 1990), like smoking or alcohol use in pregnancy, are also associated with maternal depression (Le Strat, Dubertret, & Le Foll, 2011; Marcus, Flynn, Blow, & Barry, 2003; Smedberg, Lupattelli, Mardby, Overland, & Nordeng, 2015). Antidepressants, which cross the placenta, could alter serotonin metabolism, a key neurotransmitter in early neurodevelopment (Brummelte, Mc Glanaghy, Bonnin, & Oberlander, 2017). Postnatally, parenting behavior is a key factor in the development of child self-regulation, including EF (Bernier, Carlson, Deschenes, & Matte-Gagne, 2012; Bernier, Carlson, & Whipple, 2010; Blair, Raver, & Berry, 2014; Choe, Olson, & Sameroff, 2013), and may be altered in the setting of depression through reduced sensitivity, regulatory caregiving, and engagement (Feldman et al., 2009; Vasquez-Echeverria, Alvarez-Nunez, Gonzalez, Loose, & Rudnitzky, 2022). Both periods likely have significant gene–environment interaction effects (Deater-Deckard, 2014; Friedman et al., 2008), although the magnitude of this is subject to debate (Snyder, Miyake, & Hankin, 2015).

Research links maternal depressive symptoms (MDSs) to child cognitive development (2003; Liu et al., 2017; Stein et al., 2014), and specifically EF (Power, Van Ijzendoorn, Lewis, Chen, & Galbally, 2021). However, existing studies mostly fail to distinguish depressive symptoms from diagnosed depression, with only one study using a diagnostic measure of depression (Priel, Zeev-Wolf, Djalovski, & Feldman, 2020). Depressive symptoms assessed over a shorter period or cross-sectionally do not account for what is a chronic, relapsing illness in many cases (Howard et al., 2014; O'Hara & Swain, 1996), as chronicity may contribute to developmental impact (Sohr-Preston & Scaramella, 2006). Interpretation of EF outcomes present challenges, with few valid measures for younger children, and reliance on parent-report questionnaires, which may assess social competence instead. The current study aimed to investigate the association between perinatal depression and preschool-age EF by using measures of both depressive symptoms and diagnosed depression, focusing on potential prenatal mediators.

The first part of this study evaluated maternal depressive diagnosis and perinatal depressive symptom trajectory as predictors of child EF at age 3.5–4 years. We hypothesized that women with a depression diagnosis would have a higher initial depressive symptom load that persisted longer. Depressive diagnosis and symptom growth trajectories were expected to predict EF performance at age 4. We applied standardized measures of childhood EF, independent of parental reports, thereby controlling for report bias and shared variance. We also included maternal university education as a covariate representing socioeconomic status, and a measure of maternal cognition to test for confounding effects (Hackman, Farah, & Meaney, 2010; Miyake & Friedman, 2012).

The second part assessed potential prenatal risk factors as mediators of the association between maternal depression diagnosis and child EF outcomes. While there are many theoretically relevant prenatal variables, we selected maternal smoking and alcohol use due to associations with maternal depression, and antidepressants due to the potential to alter early serotonergic metabolism in the developing brain.

## Methods

### Study design and setting

The sample was drawn from the Mercy Pregnancy and Emotional Wellbeing Study, a prospectively recruited pregnancy cohort based in Melbourne, Australia (Galbally et al., 2017). Initial recruitment was through antenatal clinics of Mercy Hospital for Women, a major metropolitan obstetric center, with assessment over six waves: wave 1 at recruitment in early pregnancy, wave 2 in the third trimester, wave 3 at delivery, wave 4 at 6 months postpartum, wave 5 at 12 months postpartum, and wave 6 at 3.5–4 years postpartum. The study protocol (Galbally et al., 2017), and details of wave 6 data collection (Galbally et al., 2020) have been published.

Ethics approval was provided by the Mercy Health Human Research Ethics Committee and all participants provided written informed consent. Mercy Health Human Research Ethics Committee, Ethics Project Number: R08/22.

### Participants

The sample included 197 women and their children who participated at wave 6, 4 years postpartum, with at least one child EF outcome measure completed.

#### Inclusion criteria

Women before 20 weeks' gestation at recruitment, booked to deliver at Mercy Hospital for Women, sufficiently proficient in English to give informed consent.

#### Exclusion criteria

Previous diagnoses of bipolar affective disorder or psychotic disorders, confirmed by Structured Clinical Interview for DSM-IV (SCID-IV). Further exclusion criteria were a history of substance abuse, intellectual disability, current involvement with child protective services, pre-existing severe physical illness, or inability to give informed consent.

### Measures

#### Maternal depressive symptoms

The SCID-IV is a widely used semi-structured diagnostic interview to determine the historical presence of psychiatric disorders meeting Diagnostic and Statistical Manual of Mental Disorders, fourth edition (DSM-IV) criteria (First, 1997), and generates diagnoses based on DSM criteria with moderate to excellent inter-rater reliability (Lobbestael, Leurgans, & Arntz, 2011). The Mood Disorders Schedule of the SCID-IV was administered at wave 1, and binary coding of 1 = current depression, and 0 = not currently depressed was used for analysis. The test was administered by trained interviewers with a sample of interviews checked for accuracy by an experienced supervisor.

MDSs at wave 1 (first trimester), wave 2 (third trimester), wave 4 (6 months postpartum), and wave 5 (12 months postpartum) were measured using the Edinburgh Postnatal Depression Scale (EPDS) (Cox, Holden, & Sagovsky, 1987). This scale has been validated for use in Australian women during the perinatal period (Boyce, Stubbs, & Todd, 1993). Internal consistency measured by Cronbach's *α* of EPDS items in each wave ranged from 0.85 to 0.92. Frequency distributions of EPDS at each wave are included in the online Supplementary material.

### Child executive function assessment

EF was assessed objectively by experienced neuropsychologists who shared training for consistency. Three age-appropriate tasks were used: NEPSY-II statue (motor inhibition), Shape School condition B (inhibition), and Shape School condition C (shifting). To account for behaviors associated with EF in the 3 months prior to assessment, the Preschool Age Psychiatric Assessment (PAPA) was administered to mothers at the time of neuropsychological evaluation.

#### NEPSY-II: statue subtask

The statue subtask of the NEPSY-II neuropsychological test battery is designed to assess EF in the preschool age group (Korkman, Kirk, & Kemp, 2007). It is a test of motor persistence and inhibition of response, with the child instructed to stand on one leg with one thumb raised and eyes closed and ignore scripted distractions. In the 3–4 year age group the test has excellent test–retest reliability (Brooks, Sherman, & Strauss, 2009; Korkman et al., 2007). It has been shown to be sensitive to clinical conditions, with children aged 3–5 with a diagnosis of attention–deficit hyperactivity disorder (ADHD) or behavioral problems scoring lower than controls (Mahone, Pillion, Hoffman, Hiemenz, & Denckla, 2005; Youngwirth, Harvey, Gates, Hashim, & Friedman-Weieneth, 2007).

#### Shape School

Shape School was developed to assess EF relating to inhibition and shifting in the preschool age group (Espy, 1997). It is presented as a story book, comprising four conditions testing core EF tasks: control, inhibit, switch, and both. For the control condition the story commences with 15 circle and square characters playing at school. The child must name characters according to color. The inhibit condition (condition B) is a Stroop task. It elaborates the story, with characters now having happy or sad faces, and the child is asked to name only the happy students. The switch condition (condition C) increases complexity, as some shapes are now wearing hats. The child must name the characters by color for those with no hat, and by shape for those wearing a hat. Condition D was not administered to this cohort. An efficiency score was calculated to use in analysis, whereby efficiency = (number of correct answers − number of errors)/total time to complete condition (Nieto, Ros, Medina, Ricarte, & Latorre, 2016). It has been shown to be sensitive to complexity of the subtest, and age at testing, suggesting that it is a responsive measure in this age group (Espy, Bull, Martin, & Stroup, 2006). Performance at 54 months has been found to predict numeracy and literacy at school entry (Bull, Espy, & Wiebe, 2008).

In this sample Cronbach's *α* was 0.83 for condition B, and 0.70 for condition C.

#### Preschool age psychiatric assessment (PAPA)

The PAPA is a structured parent interview using DSM-V symptoms to generate DSM-V diagnoses via algorithms (Egger, Angold, Small, & Copeland, 1999). Parents are asked for examples of symptoms over the previous 3 months that are coded using a glossary. Interviewers undertook authorized training to establish reliability, with a sample checked by an authorized trainer. For this study the nine items from the ADHD module, inattentive subtype, were used. The test–retest reliability for the overall ADHD module is 0.74 (Egger et al., 2006).

The PAPA relies on the DSM-V ADHD construct, which involves establishing symptoms that differ from developmental norms. This presents a challenge in early childhood when there is a broad range of accepted behaviors (Curchack-Lichtin, Chacko, & Halperin, 2014). However, inattentive symptoms and the inattentive ADHD subtype have been found to be more stable from infancy onward than hyperactivity-impulsivity symptoms (Lahey, Pelham, Loney, Lee, & Willcutt, 2005; Vergunst et al., 2019).

Cronbach's *α* in this sample was 0.73. As reflects a non-clinical child sample, a high proportion of zero scores (67.6%) was found.

### Covariates and mediators

Maternal age, maternal university education, employment status, relationship status, and antidepressant use in pregnancy were recorded at recruitment. Maternal university education was used as a proxy for socioeconomic status, consistent with many studies in child development (Desai & Alva, 1998). Child date of birth, sex, and gestational age were collected at delivery.

Heritability of EF is reported to be high. To evaluate the contribution of maternal cognition, the Test of Premorbid Function (TOPF) was used at wave 6. The test is a list of 70 irregular words of increasing difficulty in English. It has excellent internal consistency (*r* = 0.92–0.99) and test–retest stability (*r* = 0.89–0.95) (Holdnack & Drozdick, 2009), with validity established by correlation with verbal skills (Wechsler Adult Intellectual Scale-IV [WAIS-IV], *r* = 0.75) and general intellectual functioning (WAIS-IV, *r* = 0.70) (Chu, Lai, Xu, & Zhou, 2012).

Maternal smoking and alcohol use were assessed via self-report questionnaire at waves 1 and 2. Exposure to smoking at wave 1 closely correlated with response at wave 2 and an affirmative response at either timepoint was recorded as a binary variable where 0 = no exposure to smoking, 1 = any exposure to smoking. The same procedure was performed for alcohol use.

### Statistical analyses

Descriptive and bivariate analyses were conducted using SPSS version 27 (IBM Corp., 2020). Spearman correlations examined zero-order bivariate associations between observed study variables prior to testing the hypotheses using *MPlus 8* (Muthén & Muthén, 2020). Full information maximum-likelihood estimation with robust standard errors was used to handle missing data and moderate non-normal data.

A conditional latent growth curve model (LGCM) was developed to test the hypothesis that maternal depression diagnosis and symptom change during the perinatal period (i.e. illness/symptom trajectories) would predict EF outcome. LGCM was chosen over a nested design as our data were not multilevel. LGCM estimates the mean starting point (intercept factor), change (slope factor), and change on change (quadratic factor) for a study population as fixed effects and provides information about individual variance from these mean values by calculating random effects for these growth factors. Analysis proceeded in three stages: (1) sequential LGCMs were fit to determine the appropriate model of change in EPDS scores from waves 1, 2, 4, and 5 (Bollen & Curran, 2006); (2) controlling for the effect of depression diagnosis on initial symptom level and change in depressive symptoms by adding it as a predictor to these growth factors, as antenatal depression diagnosis could be expected to predict higher growth curve intercept and subsequent depressive symptom slope; and (3) EF outcomes were regressed on to these conditional growth factors and covariates representing socioeconomic status and maternal cognition. Model fit was assessed using the chi-square test with *p* < 0.05, and fit indices root mean squared error of approximation (RMSEA) <0.06, standardized root mean square residual (SRMR) <0.08, comparative fit index (CFI) >0.95, with lower adjusted Bayesian information criterion (aBIC) representing a better fitting model (Hu & Bentler, 1999).

Four EF outcomes were tested: (1) the inattentive symptom count from the PAPA ADHD subscale; (2) NEPSY statue scaled score; (3) Shape School condition B; and (4) Shape School condition C. For each outcome, the baseline model tested whether MDS growth trajectories predicted EF outcome. Second, covariates (binary maternal university education, and continuous maternal TOPF score) were added as predictors of EF outcome.

EF measures in our sample were not significantly correlated, consistent with similar studies (Doebel, 2020; Gartner & Strobel, 2021). Measures of behavioral inhibition (statue), cognitive inhibition (Shape School B), and switching (Shape School C) are therefore analyzed and considered separately. For analysis of the PAPA inattentive symptom count, the mean (0.61) was less than the variance (1.45) suggesting overdispersion, therefore a negative binomial hurdle model was used.

The second hypothesis examined whether prenatal exposure to smoking, alcohol, or antidepressants mediated an association between MDS level during pregnancy (intercept factor) and EF outcome. Each exposure was examined separately to avoid issues of collinearity between exposures, using *MPlus* to compare indirect and direct pathways.

#### Power analysis

A priori power analysis was performed based on a desired statistical power of 0.8, and a probability of 0.05. The minimum sample needed to detect a moderate effect for a structural equation model with eight predictors, including covariates, and two latent variables in the baseline model was 90, with 100 recommended minimum sample size given model complexity. A small effect would require 223 participants.

#### False-discovery rate correction

Benjamini–Hochberg false-discovery rate correction was employed (Benjamini & Yekutieli, 2005). This method is a sequential Bonferroni technique that assigns a ranking to *p* values in a model and calculates a critical *p* value based on the formula (*i*/*m*)*Q*, where *i* is the *p* value's rank, *m* is the total number of tests, and *Q* is the false-discovery rate (0.05). For example, in our sample with 32 predictors of outcome tested, the critical *p* value was 0.042. Associations that remained significant after this procedure are highlighted in bold in the relevant tables.

## Results

### Sample characteristics

Demographic data are presented in Table 1. Comparison between the original cohort (*N* = 287) and those who remained in the study at wave 6 (*N* = 197) confirmed that missing data were not selective, with no significant differences in maternal smoking and alcohol use in pregnancy, university education, antidepressant use, depression diagnosis, or maternal age. Mean birthweight was 3.49 kg (range 1.37–5.46 kg), with a mean gestational age of 39.7 weeks (range 30.3–42.1). Male infants comprised 59.8% of the sample. Mean child age at wave 6 testing was 48.9 months, with children of mothers with antenatal depression diagnosis slightly older than those without. Correlations between study variables are shown in Table 2.

**Table 1.** Maternal participant demographic information (*N* = 197)

	No depression diagnosis (*n* = 156)	Depression diagnosis (*n* = 41)	χ^2^ *p* value
**Maternal age (mean)**	32.06 (0.36)	30.05 (0.92)	0.069
Maternal education			0.13
High school	12 (7.7%)	2 (4.9%)	
Completed apprenticeship/diploma	33 (21.3%)	14 (34.2%)	
Completed bachelor's degree	66 (42.6%)	16 (39.0%)	
Completed postgraduate degree	44 (28.4%)	9 (22.0%)	
Employment			0.008
Full time work	111 (71.6%)	17 (41.5%)	
Part time/casual work	28 (18.0%)	19 (46.3%)	
Unemployed/studying	14 (9.0%)	3 (7.2%)	
Relationship status			<0.001
Not in a relationship	4 (2.6%)	7 (17.1%)	
Married or de facto	151 (97.4%)	34 (82.9%)	
Smoking quantity in pregnancy			0.427
None	134 (88.7%)	36 (87.8%)	
Less than 1 cigarette per day	3 (2.0%)	2 (4.9%)	
1–5 cigarettes per day	8 (5.3%)	1 (2.4%)	
More than 5 cigarettes per day	6 (4.0%)	2 (4.9%)	
Alcohol consumption in pregnancy			0.428
None	95 (63.8%)	27 (67.5%)	
1 standard drink or less per week	49 (32.8%)	12 (30%)	
2–6 standard drinks per week	5 (3.3%)	1 (2.5%)	
1 or more standard drinks per day	0	0	
Antidepressant use in pregnancy	15 (9.6%)	15 (36.6%)	<0.001

**Table 2.** Descriptive statistics and bivariate correlations for study variables

	*M* (s.d.)	1	2	3	4	5	6	7	8	9	10	11	12	13	14	15	16
1. Maternal age	31.64 (4.68)																
2. University education^a^	–	0.13															
3. SCID depression^b^	–	−0.14*	−0.09														
4. Antenatal antidepressant^c^	–	0.13	−0.08	0.31***													
5. Smoking in pregnancy^d^	–	0.08	−0.25***	−0.07	0.08												
6. Alcohol use in pregnancy^e^	–	0.22**	−0.03	−0.06	−0.06	0.23**											
7. EPDS wave 1	6.24 (4.43)	−0.10	−0.09	0.42***	0.16*	0.03	0.01										
8. EPDS wave 2	6.43 (4.62)	−0.15*	−0.01	0.37***	0.19**	0.03	0.08	0.73***									
9. EPDS wave 4	5.94 (4.69)	0.15*	−0.00	0.23**	0.14	0.06	0.01	0.51***	0.50***								
10. EPDS wave 5	5.74 (4.61)	−0.02	−0.07	0.38***	0.26***	0.07	−0.10	0.58***	0.59***	0.63***							
11. EPDS wave 6	5.48 (4.56)	−0.03	−0.03	0.37***	0.31***	−0.06	0.03	0.53***	0.47***	0.27***	0.53***						
12. Maternal TOPF score	55.2 (9.32)	0.02	0.38***	−0.17*	−0.05	0.05	−0.13	−0.01	−0.05	−0.04	−0.17*	−0.11					
13. PAPA inattention score	0.61 (1.2)	−0.07	−0.15*	0.12	0.04	0.13	0.16*	0.07	0.09	0.06	−0.06	−0.11	−0.11				
14. NEPSY statue scaled score	9.69 (2.93)	−0.01	−0.08	−0.01	0.11	0.10	0.14	−0.02	0.05	−0.02	−0.23**	−0.07	−0.04	−0.11			
15. Shape School condition B	13.96 (1.95)	0.24**	0.09	−0.06	−0.11	−0.14	0.05	−0.00	−0.02	−0.10	0.16*	0.07	0.08	−0.15	−0.11		
16. Shape School condition C	11.73 (3.06)	−0.01	0.28***	−0.01	−0.14	0.06	−0.04	−0.20*	−0.25**	−0.16	0.10	0.03	0.23**	−0.09	0.09	0.16	

a University education: 0 = no tertiary education, 1 = tertiary education.

b SCID depression: 0 = no depression diagnosis, 1 = depression diagnosis present.

c Antenatal antidepressant: 0 = no antenatal antidepressant prescribed, 1 = antenatal antidepressant prescribed.

d Smoking in pregnancy: 0 = no smoking reported, 1 = smoking during pregnancy.

e Alcohol use in pregnancy, 0 = alcohol used in pregnancy, 1 = no alcohol used in pregnancy.

*p < 0.05, **p < 0.01, ***p < 0.001.

Child EF scores for NEPSY-II statue and Shape School inhibit and switch conditions were similar to scores published in other non-clinical samples of children not born prematurely (Espy, 1997; Pritchard & Woodward, 2011; Wiebe, Espy, & Charak, 2008).

Where the mother completed the PAPA inattention module but the child was unable to complete one or more of the neuropsychological tasks, the Mann–Whitney *U* test showed that there was a statistically significant difference in median inattentive symptoms, with the group unable to complete all tasks having a higher mean rank PAPA symptoms than the group able to complete all EF tasks (*U* = 763, *p* = 0.035). This suggests that children identified by their mother as having more inattentive symptoms were less tolerant of objective EF testing, a point we will return to in the discussion.

### Maternal depressive symptom latent growth curve

Linear and quadratic depressive slopes were estimated, with model fit statistics reported in the online Supplementary material. Misspecification of the quadratic model was addressed by fixing the variance to 0, which resulted in a model that fit the data well but failed to specify when growth trajectories were used as predictors. Therefore, the linear model was used in analysis. When the latent growth factors were controlled for maternal depression diagnosis at wave 1 model fit improved.

Average EPDS at wave 1 was 5.58 (i.e. fixed intercept effect) for women without depression, with significant variation around this starting average (i.e. random intercept effect). Although fixed linear change (*b* = −0.10) for these women was not significantly different from zero, there was significant variation around the fixed slope. For women with a depression diagnosis, wave 1 EPDS was significantly higher by an average 4.20 symptoms. Depression diagnosis was not associated with significantly different fixed slope effects.

### PAPA inattention subscale

After false-discovery rate correction a significant, positive association was found between the MDS slope and PAPA inattention score, with an incident risk ratio of 3.19, meaning that for every 1-unit increase in depressive symptom slope from 0, there was an associated increase in inattention symptom count that was 3.19-fold higher than the baseline non-zero symptom count of 0.95. Results are shown in Table 3.

**Table 3.** Estimated coefficients and incident rate ratios (with standard error) for Maternal Depressive Symptoms and Inattentive Symptom Count.

	EF Outcome 1: Inattention Symptom Count			
	Unadjusted Model		Adjusted Model	
*Fixed Effects*	*Unstandardised Results*	*Incident Rate Ratio* (*95%CI*)	*Unstandardised Results*	*Incident Rate Ratio* (*95%CI*)
**Depression Diagnosis^a^**	−1.01 (.64)	.36(-.09-.88)	−1.40 (.75)	.25(−.11−.61)
**Depressive Symptoms Initial Score (Intercept)**	.08 (.11)	1.08 (.86−1.31)	.10 (.10)	1.10 (.88−1.32)
**Depressive Symptoms Change during Perinatal Period (Slope)**	1.2* (.40)	3.19 (.75−5.63)	1.19** (**.35)**	3.28 (1.02−5.55)
**Maternal University Education^b^**	−	−	−1.04** (**.40)**	.36 (−1.81−.26)
**Maternal Cognition^c^**	−	−	.04 (.03)	1.04 (−.01−.10)
***Random Effects***				
**Intercept**	8.24*** (1.12)	−	7.89*** (1.25)	−
**Slope**	.34*** (.09)	−	.36** (.09)	−
**aBIC**	4418.88	−	4194.84	−

* p < .05, ** p < .01, *** p < .001.

Results that remain significant after false discovery rate correction are highlighted in bold

^a^Binary variable where 0=no depression diagnosis, 1 = depression diagnosis measured antenatally using the Structured Clinical Interview for DSM-IV.

^b^Binary variable 0 = no university education, 1 = university education

^c^Measured using the Test of Premorbid Function

The addition of covariates improved model fit, and the slope factor remained significant. Maternal university education was also a significant predictor of child inattention symptom count (*B* = −1.04, *p* < 0.009, Incident Rate Ratio = 0.36, 95% confidence interval [CI] 0.08–0.63). It was associated with an approximate 70% reduction in the baseline non-zero inattention symptom count of 0.95, meaning that mothers with a university education were less likely to report inattention symptoms in their children. This model explained only a small amount of the variance in child inattention symptoms, with pseudo-*R*^2^ = 0.02.

### Statue scaled score: motor inhibition

Results for the statue subtest of the NEPSY-2, and two subtests of the Shape School task are presented in Table 4. A significant negative association after false-discovery rate correction was found between MDS slope and statue scaled score (*β* = −0.98, *B* = −0.20, *p* = 0.025), meaning that for every 1-unit increase in MDS gradient, there was a −0.98 decrease in statue performance from a mean of 9.67. When covariates were added this association remained significant but attenuated. The adjusted model explained only a small amount of the variance, with *R*^2^ = 0.03.

**Table 4.** Standardised coefficients (with standard error) for Maternal Depressive Symptoms and Neuropsychological Child Executive Functions Measures, unadjusted and adjusted for covariates.

	Shape School Condition B (Inhibition)		Shape School Condition C (Switching)		NEPSY Statue (Motor Inhibition)	
	Unadjusted	Adjusted	Unadjusted	Adjusted	Unadjusted	Adjusted
***Fixed Effects***						
**Depression Diagnosis^a^**	.02 (.10)	.04(.09)	.06(.09)	.01(.09)	−.05(.11)	−.05(.12)
**Depressive Symptoms Initial Score (Intercept)**	−.09 (.10)	−.09 (.09)	−.17 (.12)	−.04 (.12)	.12 (.12)	.05(.13)
**Depressive Symptoms Change during Perinatal Period (Slope)**	0.06 (.09)	.05 (.10)	−.19* (.09)	−.19* (.09)	−.20* (**.09)**	−.14(.10)
**Maternal University Education^b^**	−	.09 (.10)	−	.18* (.09)	−	.06 (.10)
**Maternal Cognition^c^**	−	−.06 (.07)	−	.14 (.07)	−	−.06 (.12)
***Random Effects***						
**Intercept Factor**	.69*** (.08)	.67*** (.09)	.69*** (.08)	.69*** (.09)	.69*** (.08)	.67*** (.09)
**Slope Factor**	.99*** (.01)	.99*** (.01)	.99*** (.01)	.99*** (.01)	.99*** (.01)	.99** (.01)
**aBIC**	4731.23	4506.72	4746.35	4496.69	4856.09	4607.03
**R-square**	.01 (.02)	.02 (.02)	.06 (.05)	.10* (.04)	.05 (.04)	.03 (.03)

* p < .05, ** p < .01, *** p < .001.

Results that remain significant after false discovery rate correction are highlighted in bold

^a^Binary variable where 0 = no depression diagnosis, 1 = depression diagnosis measured antenatally using the Structured Clinical Interview for DSM-IV.

^b^Binary variable 0 = no university education, 1 = university education

^c^Measured using the Test of Premorbid Function

### Shape School condition B: cognitive inhibition

There were no statistically significant relationships between diagnostic depression, MDS growth trajectories, and cognitive inhibition, as measured by Shape School condition B performance. While adding covariates to the model improved model fit, they did not predict inhibition.

### Shape School condition C: switching

A statistically significant association was found between MDS slope and condition C performance that remained significant when covariates were included in the model (*β* = −0.93, *B* = −0.19, *p* = 0.04), suggesting that the more depressive symptoms increased across the perinatal period, the poorer performance on this task. Maternal university education was a significant predictor of condition C score in the adjusted model (*B* = 0.18, *p* = 0.05), with children showing better performance if their mother had a university education when controlling for MDS growth. The *p* values in this model did not remain significant when false-discovery rate correction was performed.

### Indirect pathways via prenatal exposures

No statistically significant direct or indirect pathways were found via these prenatal exposures and child EF outcomes measured. Path estimates are shown in Tables 5. It should be noted that models predicting inattentive symptom count via maternal smoking and alcohol did not identify, potentially due to low rates in the sample.

**Table 5.** Direct and indirect path estimates from maternal antenatal depression diagnosis to child executive function outcomes via prenatal exposure of maternal smoking, alcohol, and antidepressant use

Child EF Outcome	Mediator	Path Estimate	
		Direct	Indirect
**Shape School Condition B**	Smoking	−.13	.00
	Alcohol	−.11	−.02
	Antidepressant	.10	−.28
**Shape School Condition C**	Smoking	−.03	.00
	Alcohol	−.04	.01
	Antidepressant	.20	−.34
**NEPSY Statue**	Smoking	.16	−.00
	Alcohol	.16	−.01
	Antidepressant	.36	−.19
**Inattentive Symptom Count**	Smoking		
	Alcohol		
	Antidepressant	−.35	.22

* p < .05, ** p < .01, *** p < .001.

Note: the models for inattentive symptoms mediated via prenatal smoking and alcohol exposure, likely due to low rates in the sample

## Discussion

There are four key findings of this study. First, the persistent and increasing trajectory of MDSs over the perinatal period was associated with poorer EF outcomes in children at age 4. It suggests salience of chronicity and severity of maternal depression over the perinatal period – rather than the diagnostic threshold of a depressive illness – in predicting EF outcome. Second, the maternal depressive trajectory, more specifically, predicted child motor inhibition and inattentive symptoms after false-discovery rate correction when controlling for diagnosed depression. Third, the association with inattentive symptoms remained after adjustment for covariates of maternal cognition and university education. Fourth, prenatal exposure to smoking, alcohol, and antidepressants did not mediate pathways from MDSs to EF outcomes. However, outcome or mediation effects of smaller effect size may not be detected due to the relatively small sample size.

This study provides preliminary support for an association between perinatal MDS and EF deficits in children at age 4. Specifically, this association was detected in the perinatal MDS trajectories (likely indexing chronic, severe, or ascending course of depressive symptoms) rather than a depression diagnosis. The impacts were detected by both (i) objective measures of motor inhibition, and (ii) parental report of symptoms regarding inattentive behaviors over the previous 3 months as assessed by gold-standard research structured interview. This means that when MDS increased across the perinatal period, children performed more poorly on these EF measures and their mothers were more likely to report increased inattentive symptoms. For two measures, PAPA inattention score and switching, maternal university education was a statistically significant predictor of the outcome, supporting evidence of the importance of socioeconomic status in the development of EF in children (Hackman, Gallop, Evans, & Farah, 2015).

These findings may indicate that the ascending trajectory of symptoms in later pregnancy or the year postpartum is relatively more important in EF development. This may reflect the importance of timing of MDS (i.e. exposure window effects), or chronicity (i.e. dose–response effect) as more impactful. While our previous meta-analysis did not find a difference between prenatal and postnatal exposure to MDS in terms of child EF outcome, some studies examining both prenatal and postnatal periods do show that the postnatal period and beyond are more potent indicators (Hutchison, Mâsse, Brain, & Oberlander, 2019; Jensen, Dumontheil, & Barker, 2014; Nolvi et al., 2018; Park, Brain, Grunau, Diamond, & Oberlander, 2018; Rotheram-Fuller et al., 2018). A recent large population study using latent class analysis found that chronicity of MDSs over pregnancy and early childhood predicted child inhibitory performance, but those exposed to MDSs in the prenatal period alone did not show impaired EF (Choe, Deer, & Hastings, 2023). This evidence, added to our findings, suggests that chronic or postnatal MDSs are more important drivers of child EF development, possibly due to altered maternal behavior in infancy and early childhood.

Some caution is needed in interpreting these findings, as the association with motor persistence was not evident after adjusting for covariates. For the switching task, false-discovery rate correction suggested that *p* value criteria were not met. Additionally, our models explained only a small proportion of the variance in outcome measures. However, it is possible that our data underestimated the true relationship, as children with the highest burden of inattentive symptoms – expected to perform poorly on objective testing – were least likely to complete neuropsychological assessment. Adding to this, the association with PAPA inattention score – completed by all dyads in the study – remained significant after addition of covariates and false-discovery rate correction. Shared variance could contribute here, as mothers with depression are more likely to report negative behaviors in their children, but was mitigated through use of a structured, validated instrument with trained assessors. Additionally, the a priori power analysis suggested that we were slightly below the sample size required to detect a small effect size. This trade-off between measurement rigor and sample size is a frequent practical limitation; the only other study to use a maternal diagnostic measure in investigating child EF was similarly sized (Priel et al., 2020). Our findings are more hypothesis-generating (i.e. suggestive) rather than hypothesis-testing (i.e. conclusive) and await future replication.

Children with inattentive symptoms in our study were less likely to complete age-specific objective assessments, highlighting testing difficulties. Younger children's test performance is influenced by environmental test conditions, sleep, stimulation, and task motivation (Anderson & Reidy, 2012; Lillard, Drell, Richey, Boguszewski, & Smith, 2015; Turnbull, Reid, & Morton, 2013). Motivation to perform forms part of EF development (Doebel, 2020), which may link ‘hot’ and ‘cool’ EF functions considering the task's emotional aspect (Zelazo & Carlson, 2012). Future EF measurements can address this deficit, facilitating understanding of developmental trajectories. The problem of weakly correlated EF measures, found in previous studies (Dang, King, & Inzlicht, 2020; Wu, Jalapa, Han, Tawfiq, & Cui, 2022), can be addressed by examining separated measures, avoiding confounding and conceptual heterogeneity, and serving transparency.

There are some additional limitations. Maternal parenting behavior, potentially affected by depressive symptoms, was not included in this analysis. We did not gather information about other family members in terms of parenting or mental health, although more limited evidence is available; to date paternal depression has been shown to have a lower impact on EF development (Vänskä et al., 2017). Our sample was also relatively higher SES and therefore missed more vulnerable populations, with a higher proportion of mothers with university-level education which could be expected to bias results in the opposite direction. We did not collect information about maternal inattention or ADHD, which may be of importance given the high heritability of ADHD (Faraone & Larsson, 2019).

### Future directions

Important influences on EF development during the postpartum period and early childhood include the parent–child relationship. Repeat measurement of EF would allow exploration of developmental trajectories in this group.

## Conclusion

This study builds on knowledge of the effects of MDS on child development by using longitudinal study design and well-validated tools to establish maternal depressive diagnosis and child EF outcome. Increasing MDS over the perinatal period was associated with increased maternally reported inattentive symptoms when controlling for maternal education and cognition. An association with motor inhibition was detected but did not remain significant when covariates were added. Socioeconomic status was associated with inattentive symptoms and switching performance, supporting established research. To our knowledge, this is the first study which has demonstrated the importance of perinatal MDS trajectory impacting child EF outcomes.

## Supporting information

Power et al. supplementary material Power et al. supplementary material

## Supplementary material

The supplementary material for this article can be found at https://doi.org/10.1017/S0033291723002118.
