# Supplementary material for: The trajectory of maternal perinatal depressive symptoms predicts executive function in early childhood
Source: Psychol Med. 2023 Oct 2;53(16):7953–63. doi: 10.1017/S0033291723002118 (PMC10755237; doi:10.1017/S0033291723002118)
Supplement: Power et al. supplementary material [file S0033291723002118sup001.docx]

**Supplemental Figure 1: Frequency distributions of Edinburgh Postnatal Depression Scale scores**


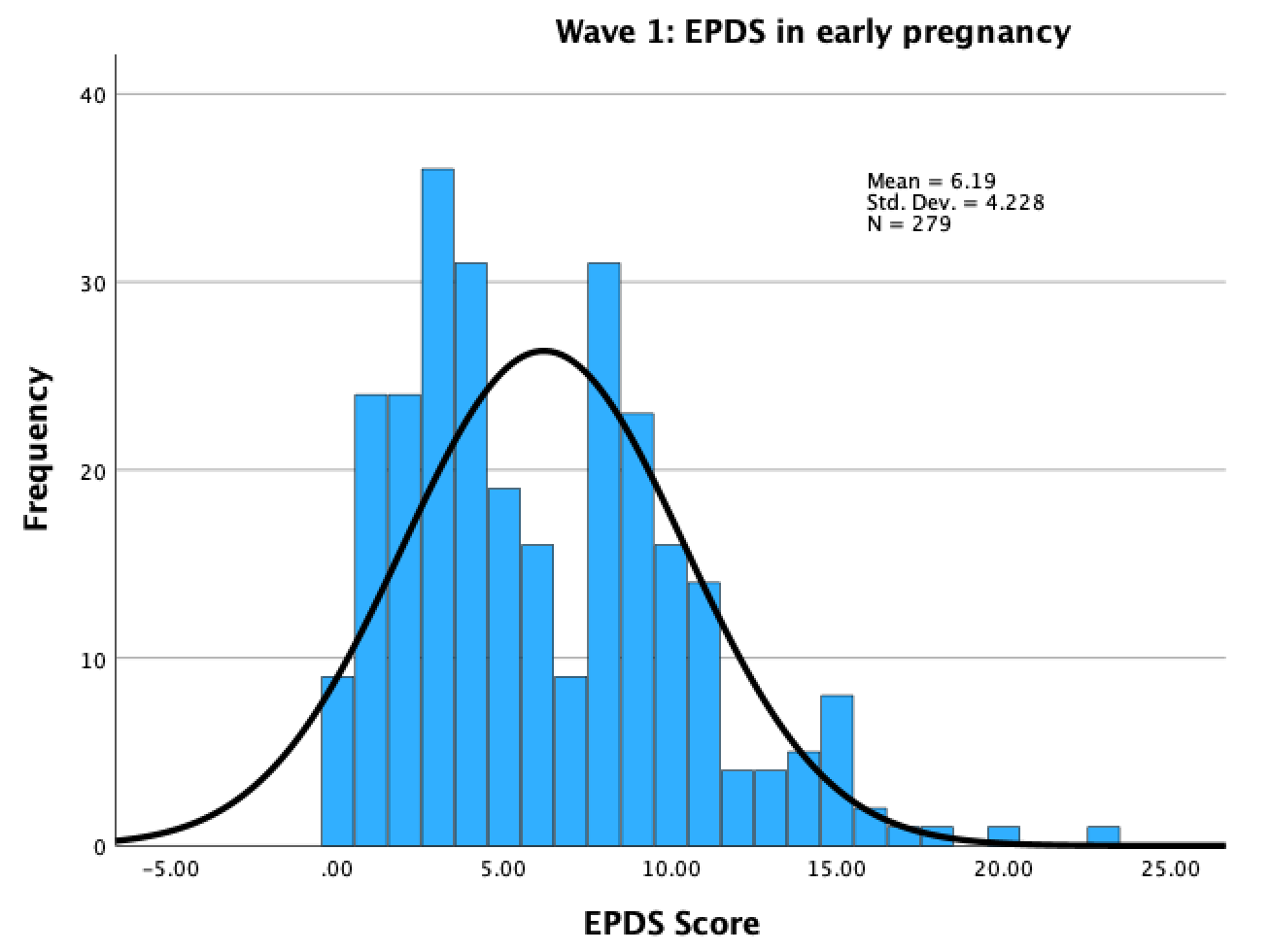

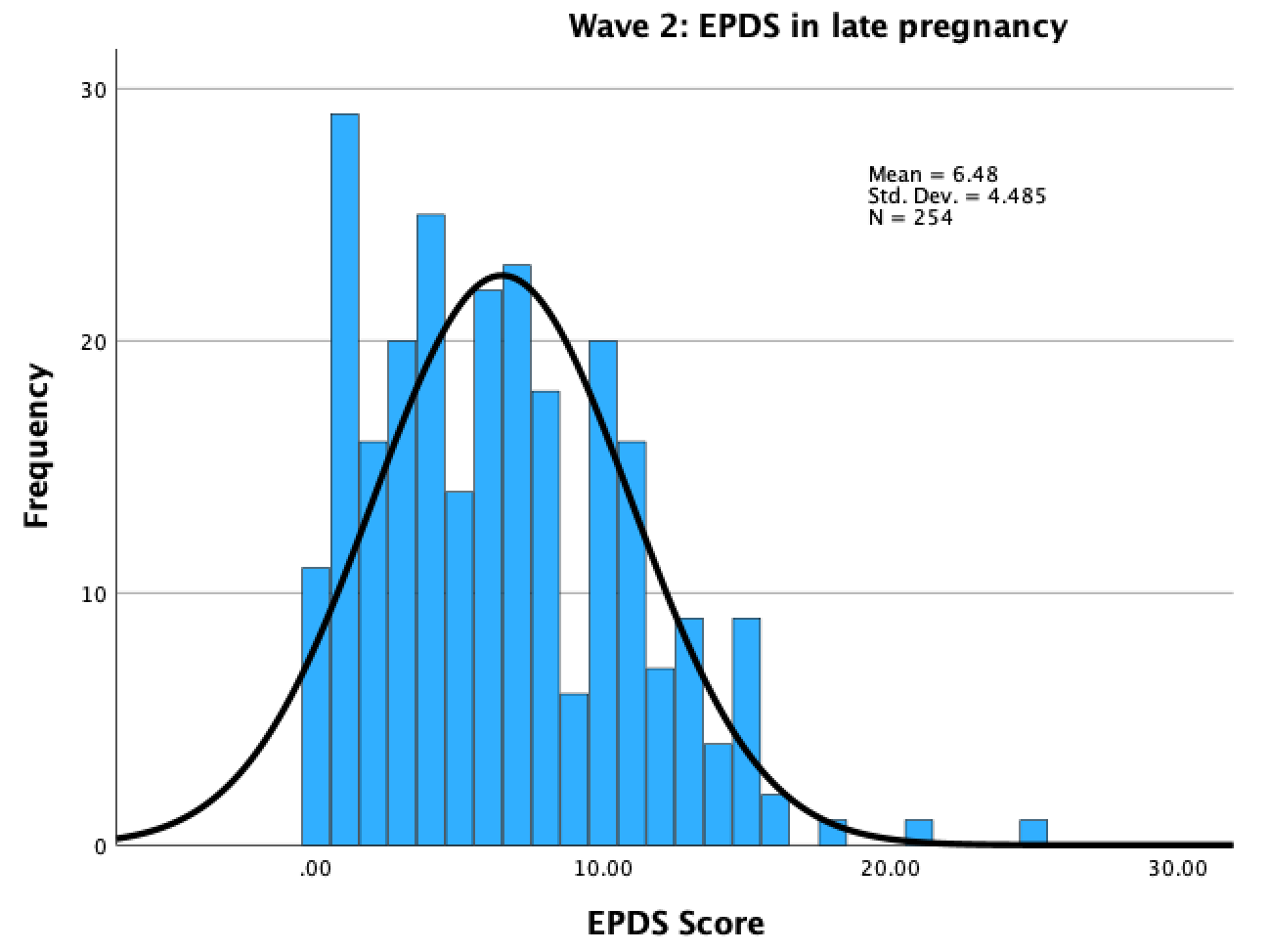


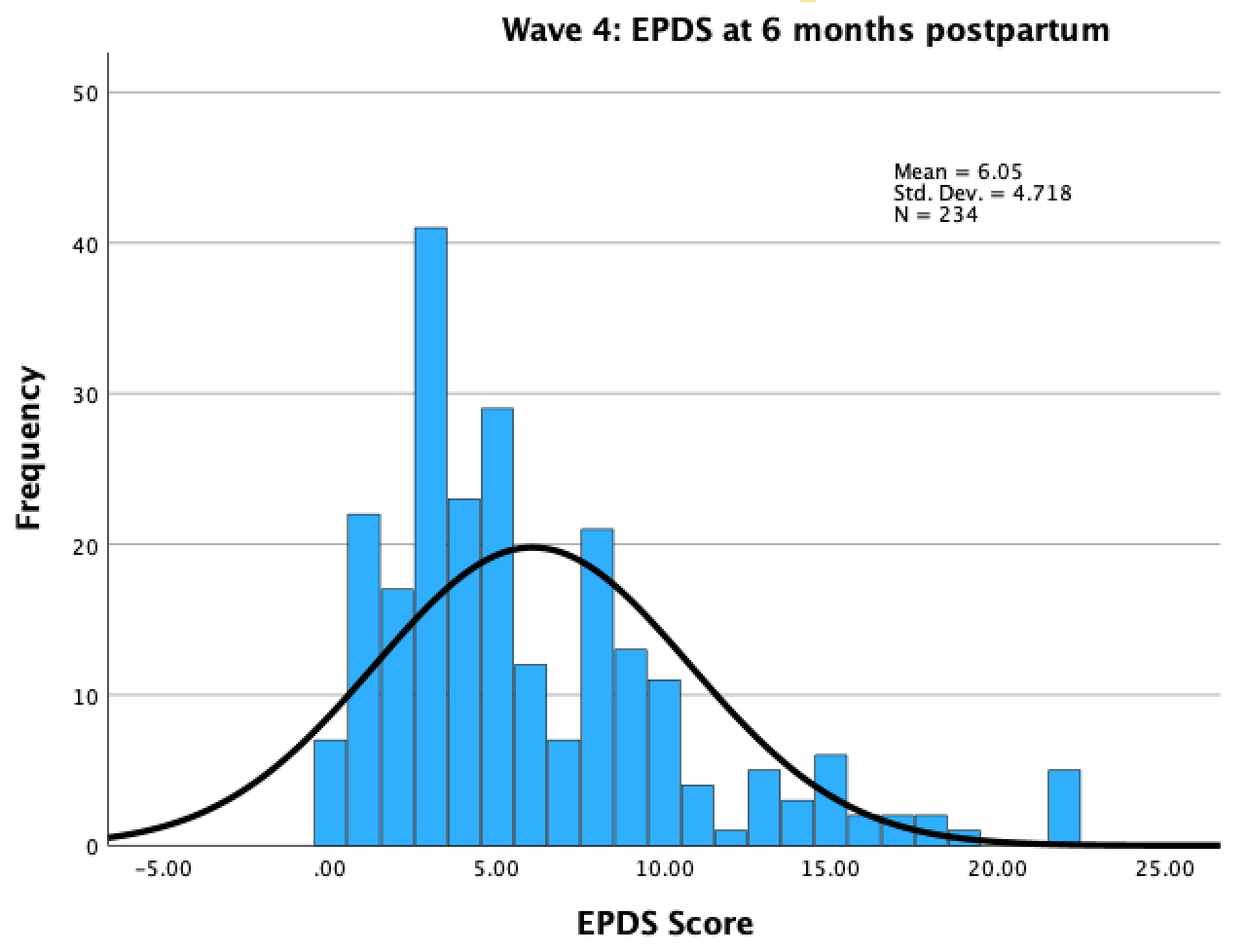

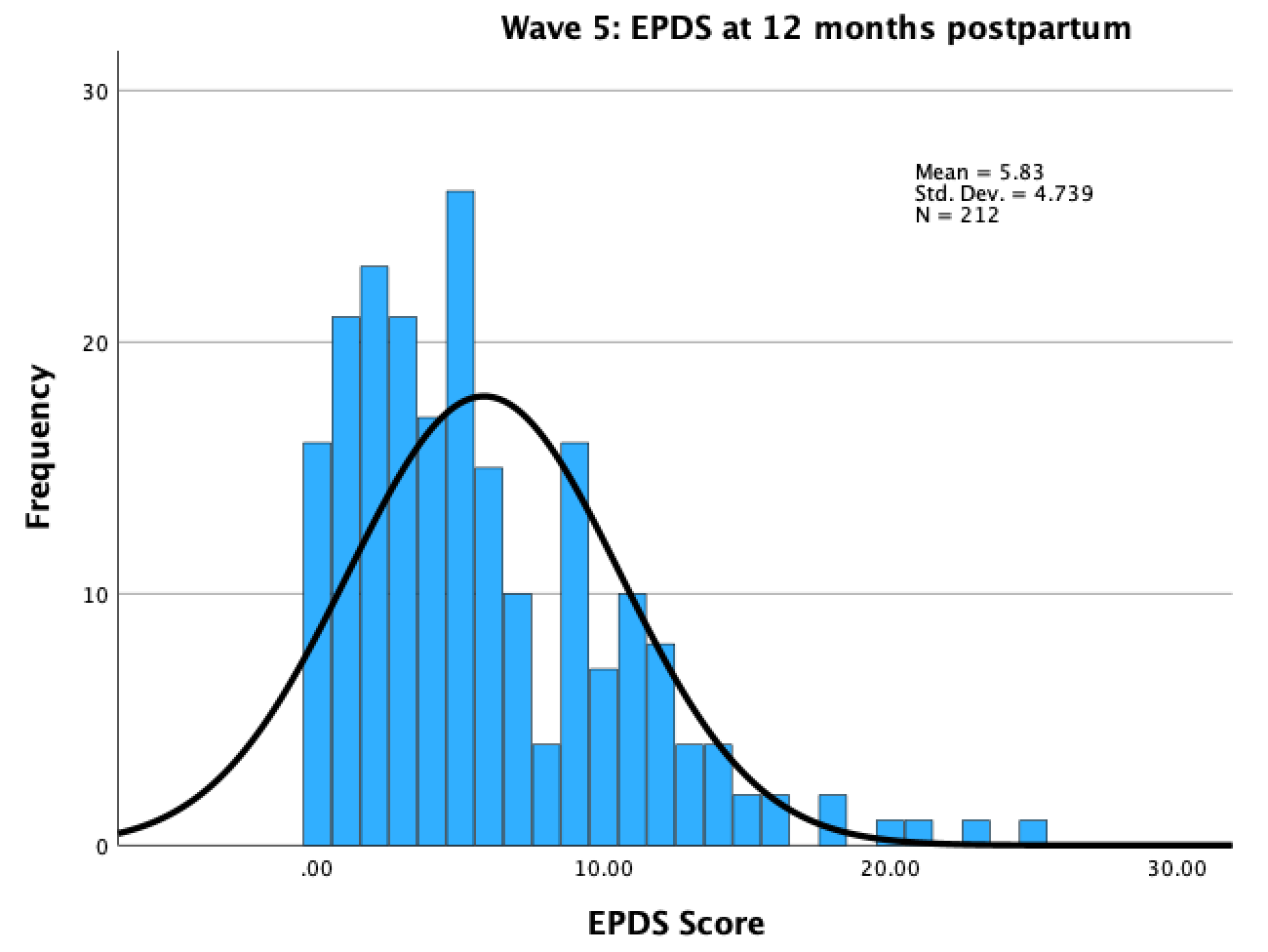


**Supplemental Table 1:**

***Fit Indices for Latent Growth Curve in Maternal Depressive Symptoms Models.***

| **Maternal Depressive Symptoms Models** | ***aBIC*** | ***Χ^2^*** | ***d.f.*** | ***p*** | ***CFI*** | ***RMSEA (95% CIs)*** |
| --- | --- | --- | --- | --- | --- | --- |
| **Intercept-only** | 4184.26 | 49.62 | 8 | 0 | 0.868 | .16 (.12, .21) |
| **Linear Slope** | 4150.67 | 9.17 | 5 | 0.102 | 0.987 | .07 (.0, .13) |
| **Quadratic** | 4149.73 | 0.28 | 1 | 0.598 | 1.00 | .06 (.00, .15) |
| **Conditional Growth Model** | 3826.26 | 4.81 | 7 | 0.684 | 1.00 | .00 (.00, .7) |
